# Identification of Enhanced Cyclooxygenase-2 (COX-2) Inhibitors Beyond Curcumin Through Virtual Screening to Target Inflammation-Related Metabolic Complications

**DOI:** 10.3390/ijms27041624

**Published:** 2026-02-07

**Authors:** Marakiya T. Moetlediwa, Rudzani Ramashia, Mpatla B. Mangale, Carmen Pheiffer, Babalwa U. Jack, Elliasu Y. Salifu, Pritika Ramharack

**Affiliations:** 1 Non-Communicable Disease Research Unit, South African Medical Research Council, Tygerberg 7505, South Africa; carmen.pheiffer@mrc.ac.za; 2 Biomedical Research and Innovation Platform, South African Medical Research Council, Tygerberg 7505, South Africa; rudzani.ramashia@mrc.ac.za (R.R.); babalwa.jack@mrc.ac.za (B.U.J.); elliasuyakubu@gmail.com (E.Y.S.); pritika.ramharack@mrc.ac.za (P.R.); 3 Department of Human Biology, Faculty of Health Sciences, University of Cape Town, Observatory 7925, South Africa; 4 Centre for Cardio-Metabolic Research in Africa, Division of Medical Physiology, Faculty of Medicine and Health Sciences, University of Stellenbosch, Tygerberg 7505, South Africa; 5 Department of Integrative Biomedical Sciences, Faculty of Health Sciences, University of Cape Town, Observatory 7925, South Africa; mngmpa002@myuct.ac.za; 6 Department of Obstetrics and Gynaecology, Faculty of Health Sciences, University of Pretoria, Pretoria 0001, South Africa; 7 Department of Pharmaceutical Sciences, Tshwane University of Technology, Private Bag X680, Pretoria 0001, South Africa; 8 Pharmaceutical Sciences, School of Health Sciences, University of KwaZulu-Natal, Westville Campus, Durban 4001, South Africa

**Keywords:** Cyclooxygenase-2, Curcumin, pharmacophore modeling, molecular docking, molecular dynamic simulations

## Abstract

Cyclooxygenase-2 (COX-2) is a key enzyme in inflammatory pathways and serves as a therapeutic target in the treatment of inflammation-related diseases. Curcumin, a bioactive polyphenol from turmeric, has gained scientific attention due to its potent anti-inflammatory properties, largely mediated through COX-2 inhibition. However, the poor solubility and limited bioavailability of Curcumin limit its potential as a therapeutic agent targeting inflammatory diseases. We used an in silico approach to identify Curcumin-like scaffolds as novel COX-2 inhibitors with improved drug-like properties and therapeutic potential. A pharmacophore model derived from the key binding moieties of Curcumin was used to virtually screen the ZINC-22 database, identifying 237 candidate compounds for further evaluation. Molecular docking further prioritized these compounds to 10 candidates with the highest binding affinities. Most hits obeyed Lipinski’s rules, except for ZINC32605424 and ZINC47133707, which exhibited high LogP and molecular weight, respectively. Toxicity screening indicated that ZINC47133693 and ZINC09499196 exhibited high safety profiles, with ZINC15942488 being highly toxic. Furthermore, certain hits such as ZINC32605424 and ZINC15942488 were predicted to be P-glycoprotein substrates and potential inhibitors of cytochrome P450. Molecular dynamics simulations confirmed the stability of COX-2–ligand complexes, with critical interactions observed at conserved residues Tyr323 and Leu320. Binding energy calculations identified ZINC32605424 as the strongest COX-2 binder, mainly stabilized by Van der Waals forces. Overall, compounds such as ZINC32605424, ZINC08644750, ZINC47133693, and ZINC09499196 demonstrated potent COX-2 inhibition. These candidates show strong potential for further preclinical validation in studies investigating inflammation-related metabolic complications.

## 1. Introduction

Chronic inflammation is characterized by persistent immune activation and dysregulated signaling pathways that play a central role in the pathogenesis of various diseases, including diabetes, cancer, cardiovascular diseases, and obesity, and various metabolic syndromes, including insulin resistance and oxidative stress [1,2,3]. Cyclooxygenase-2 (COX-2), a complex protein with distinct domains, including a small epidermal growth factor (EGF)-like domain, a membrane-binding domain, and a large globular domain containing the catalytic site [4,5], is central to the inflammatory response and rapidly upregulated in response to pro-inflammatory stimuli, growth factors, and cytokines [6]. COX-2 catalyzes the rate-limiting conversion of arachidonic acid to prostaglandins, bioactive lipids that are involved in cardiovascular, immunological, and neurological functions [1,7]. Prostaglandin biosynthesis is closely associated with inflammation. Excess prostaglandins cause redness, swelling, heat, and pain by increasing blood flow; they also activate nerves. In chronic inflammation, prostaglandins sustain immune signals, recruit inflammatory cells, and promote tissue changes like fibrosis, driving long-term inflammation [8,9]. Therefore, understanding the mechanistic role and regulation of COX-2 is important for the development of specific inhibitors to mitigate inflammation.

COX-2 is targeted by a diverse array of non-steroidal anti-inflammatory drugs (NSAIDs), which possess functional groups such as arylacetic acids, arylpropionic acids, β-ketoenols, and diarylheterocycles [10]. These NSAIDs competitively bind to the active site of COX enzymes, thereby reducing prostaglandin biosynthesis and mitigating inflammation [11]. The commonly used NSAIDs, including Sulindac, Dexamethasone, Naproxen, Aspirin, and Ibuprofen, exhibit high binding affinity and great COX-2 inhibitory activity [12]. However, these NSAIDs have adverse effects, including gastrointestinal complications, cardiovascular risks, and renal impairment. They also increase infection and bone fracture risks, especially in older adults, patients with ulcer history, or those on high doses or multiple NSAIDs [13]. This has prompted further exploration of novel COX-2 inhibitors as potential therapeutic agents [14].

In recent years, natural compounds have received increased scientific attention for their potential health benefits [15,16]. Curcumin, a bioactive compound isolated from the rhizome of the Turmeric plant, scientifically known as Curcuma longa and belonging to the Zingiberaceae family, has been reported to have various beneficial metabolic effects including anti-inflammatory properties [17,18], such as suppressing the expression of COX-2 [19]. In silico evidence has demonstrated that Curcumin has a high affinity for COX-2 [20,21]. Despite promising therapeutic benefits, the poor solubility and bioavailability of Curcumin compromise its clinical applications [22,23]. To overcome these challenges, studies have focused on assessing compounds with key functional groups present in the structure of Curcumin, but with improved inhibitory effects on COX-2.

Previously, we reported that both synthetic and natural derivatives of Curcumin have greater potential to mitigate obesity-related inflammation compared to Curcumin [21,24]. Curcumin analogs have also been shown to exhibit enhanced bioavailability [22,23,25,26,27]. Therefore, compounds retaining the key functional groups of Curcumin may serve as promising scaffolds for the identification of potent COX-2 inhibitors with high efficacy [28]. The aim of this study is to identify potential COX-2 inhibitors with structural similarities to Curcumin, but with superior molecular interactions and metabolic properties, to more effectively target COX-2 using a computational approach.

## 2. Results and Discussion

### 2.1. Pharmacophore-Based Virtual Screening for Hit Compounds

**Figure 1:**
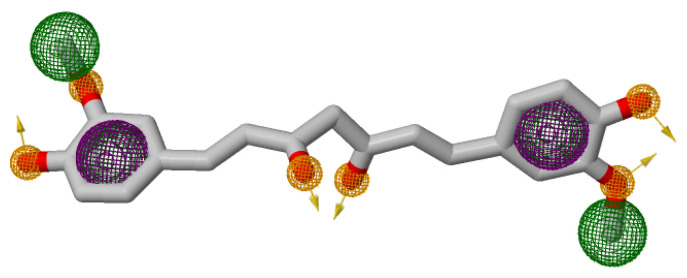
Pharmacophore model from Curcumin-COX-2 MD simulation. Colored spheres (green spheres: H-bond donors; orange spheres: hydrophobes; orange arrows: H-bond acceptors; purple: aromatics) highlight key Curcumin moieties used for ZINC-22 screening.

A pharmacophore structure shows the organization of key molecular properties involved in a ligand–receptor interaction [29]. Figure 1 illustrates the generation of a pharmacophore model, which highlights the key structural moieties interacting with Curcumin [21]. The PRED-based pharmacophore consisted of five key features: two hydrogen bond donors, two hydrogen bond acceptors, and a hydrophobic centroid. Feature placement was guided by per-residue decomposition analysis of the Curcumin–COX-2 complex, where Tyr323, Leu320, Val317, Ser321, and Arg89 contributed significantly to binding energy. These residues consistently stabilized Curcumin during the MD simulations, forming the structural basis for the pharmacophore hypothesis.

Following the pharmacophore screening, a library of 237 hits was generated and subjected to site-specific molecular docking within the binding pocket of COX-2. Molecular docking enables the prediction and analysis of how small molecules (potential drugs) interact with target proteins at the atomic level. This computational technique helps to identify candidate drugs with the most favorable binding conformations and orientations (poses) within the active site of the biological target [30].

### 2.2. Molecular Docking of Naproxen, Curcumin, and the Selected Hit Compounds

**Figure 2:**
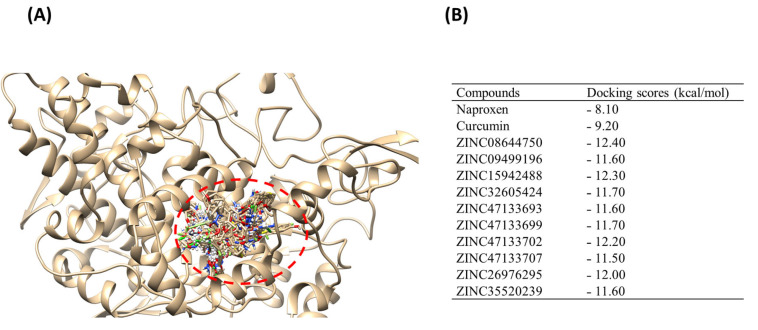
(**A**) Superimposition of ligands (Curcumin, Naproxen, and ZINC hits: distinct colors per compound) in COX-2 active site (represented as ribbon structure: tan color). (**B**) Docking scores (kcal/mol). Red broken circle: binding site pocket; ligand colors: different docked compounds.

Molecular docking was carried out to predict the binding affinity and orientation of all 237 hits. Generally, the more negative docking score suggests a stronger binding affinity [31,32]. Compounds were ranked based on their binding affinities, and the top 10 hits were selected using a cut-off docking score range between −11.50 kcal/mol and −12.40 kcal/mol, as these demonstrated stronger binding affinities compared to the reference Naproxen (−8.10 kcal/mol) and comparator Curcumin (−9.20 kcal/mol). The top 10 hits, Naproxen, and Curcumin docked within the same binding pocket on COX-2, as depicted by the superimposition illustrated in Figure 2A. Redocking of these native ligands into the active site of the COX-2 ensured the reliability of the chosen docking program. Figure 2B shows the binding scores for the top 10 hits, Naproxen and Curcumin.

**Figure 3:**
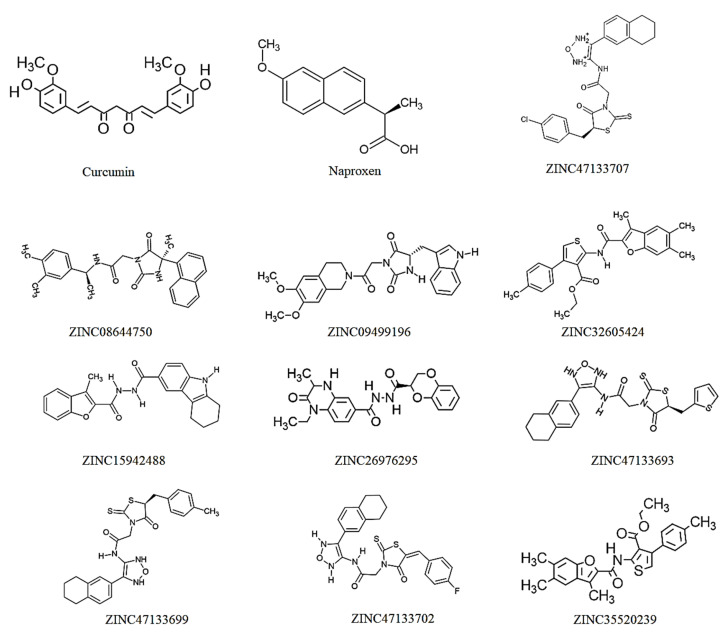
Chemical structures of Curcumin, Naproxen, and the top 10 selected hit Compounds determined by their docking scores.

Hits targeting COX-2 revealed binding affinities ranging between −11.50 kcal/mol for ZINC47133707 and −12.40 kcal/mol for ZINC0864475. The reference “Naproxen” and comparator “Curcumin” exhibited the lowest binding score values of −8.10 kcal/mol and −9.20 kcal/mol, respectively. According to docking studies [31,32], these results indicate that the chosen hits have the potential to bind COX-2 more effectively than both the comparator, Curcumin and the reference, Naproxen. The docking score of the hit compounds is related to their degree of structural similarities [33]. Compounds that closely align with the structural features and functional groups are more likely to exhibit similar docking scores. Based on these docking results, it can be deduced that the binding affinities of the selected hits, barring key functional groups found in Curcumin, were attributed to the presence of a various phenolic groups, as illustrated in Figure 3.

### 2.3. Identification of the Physicochemical Profile of Naproxen, Curcumin, and the Selected Hit Compounds

Determining whether a compound adheres to Lipinski’s rules is important in drug development as it guides the prediction of potential compounds to be oral drug candidates. These rules serve as guidelines for assessing the drug-likeness of a molecule based on its physicochemical properties, such as molecular weight, number of hydrogen bond acceptors, number of hydrogen bond acceptors, and octanol/water partition coefficient [34]. Compounds that meet Lipinski’s criteria are more likely to have favorable pharmacokinetic properties and oral bioavailability [35], increasing their chances of success in drug development. According to Lipinski’s rules, an ideal drug candidate typically meets the following criteria: a molecular weight of 500 Daltons or less, no more than 5 hydrogen bond donors, no more than 10 hydrogen bond acceptors, and an octanol–water partition coefficient (log P) of 5 or lower [21]. However, it is important to note that Lipinski’s rule is not an absolute rule but should be used as a general guide. Other factors, such as the target and intended use of the drug can also influence its drug-likeness. Physicochemical properties, such as lipophilicity, water solubility, molecular weight, and polarity, influence the absorption, distribution, metabolism, and elimination of a drug in the body [22].

This study assessed the drug-likeness of the top 10 selected hits to unveil their therapeutic potential. ZINC32605424 and ZINC47133707 did not fully comply with Lipinski’s criteria (Table 1). The ZINC32605424 and ZINC35520239 exhibited LogP values of 6.90 and 6.00, respectively, exceeding the recommended threshold; this suggests poor aqueous solubility. This high lipophilicity can cause absorption challenges because drugs that are too lipophilic tend to dissolve poorly in water, limiting their dissolution in gastrointestinal fluids and thus reducing bioavailability. ZINC47133707 had a molecular weight of 517.06 g/mol, slightly above the 500 g/mol threshold, indicating a possible reduction in permeability and bioavailability. These parameter violations may negatively impact pharmacokinetic properties and overall drug-likeness, thereby limiting their potential effectiveness as orally administered drugs.

These limitations also present opportunities for structural modification to enhance drug-likeness. For compounds with poor oral bioavailability, alternative routes of administration, such as intravenous delivery may be explored. Structural modifications could be employed to improve pharmacokinetic properties, enhance oral availability, or adapt the compounds for targeted therapeutic applications in disease contexts beyond the scope of the initial screening.

Synthetic accessibility is a measure of how easily a compound can be synthesized, with a score of 0 indicating easy to synthesize and 10 difficult to synthesize [36]. Naproxen and Curcumin exhibited synthetic scores of 1.85 and 2.97, respectively, whereas the screened ZINC compounds showed synthetic scores ranging from 3.68 to 4.86. These findings indicate that Naproxen and Curcumin may be easier to synthesize compared to the ZINC compounds. As shown in Table 1, ZINC47133699, ZINC47133707, and ZINC47133693 are predicted to be relatively more challenging to synthesize, with synthetic scores of 4.86, 4.73, and 4.69, respectively. These results suggest that while some of the screened compounds show promising drug-like properties, their synthetic complexity due to their structural moieties may pose a barrier to rapid development. In contrast, the lower synthetic complexity of Curcumin and Naproxen, together with their compliance with Lipinski’s rules, demonstrates their practicality as reference standards in this study. Overall, evaluating both drug-likeness and synthetic feasibility is critical in balancing pharmacological potential with real-world development constraints.

### 2.4. Toxicity Assessment and Physicochemical of Naproxen, Curcumin, and the Selected Hit Compounds

Clinical trials involve significant investment of time, resources, and human participation [37]. Without prior knowledge of the pharmacokinetic properties of the compound, there is a high risk of adverse effects or inefficacy, potentially harming participant safety and undermining research ethics. Computational assessment of pharmacokinetic profiles is therefore indispensable, offering a cost-effective and time-efficient means to identify promising drug candidates prior to laboratory testing. By predicting properties such as toxicity class, 50% lethal dose (LD_50_), and associated toxicity endpoints, these approaches enable early evaluation of compound safety and support the rational selection of lead candidates with favorable pharmacokinetic characteristics. In this study, the potential toxicity of hit compounds was determined using the Protox-II predictive webserver, with results summarized in Table 2.

Toxicity classification is a categorization system that ranks substances based on their potential to cause harm, with lower class numbers indicating higher toxicity [38,39]. As shown in Table 2, Naproxen and ZINC15942488 demonstrated the highest predicted toxicity (class 3). Curcumin, ZINC08644750, ZINC32605424, ZINC47133699, ZINC47133702, ZINC47133707, ZINC26976295, and ZINC35520239 belonged to toxicity class 4, while ZINC47133693 exhibited the lowest predicted toxicity (class 6). Toxicity class 3 compounds are associated with mild to moderate toxicity, with LD_50_ values ranging from 50 to 300 mg/kg for oral administration [38]. This suggests that ZINC15942488 (LD_50_: 220 mg/kg) and Naproxen (LD_50_: 240 mg/kg) may be harmful when administered orally at low doses. On the other hand, Class 4 compounds such as Curcumin (LD_50_: 2000 mg/kg), ZINC08644750 (LD_50_: 570 mg/kg), ZINC32605424 (LD_50_: 2000 mg/kg), ZINC47133699 (LD_50_: 1600 mg/kg), ZINC47133702 (LD_50_: 350 mg/kg), ZINC47133707 (LD_50_: 1600 mg/kg), ZINC26976295 (LD_50_: 1300 mg/kg), and ZINC35520239 (LD_50_: 1190 mg/kg) are generally predicted to have lower toxicity concerns at moderate doses. The observed toxicity classification of Naproxen may also account for its reported adverse effects [13].

The toxicity classification of compounds is directly correlated with their LD_50_ values, as classification is based on the lethal potential of drug candidates [40,41]. LD_50_, expressed in mg/kg of body weight, refers to the dose required to cause death in 50% of a test population (usually animals); a lower LD_50_ value indicates higher acute toxicity [42]. It should be noted that Protox-II toxicity classifications and LD_50_ values are model-based predictions derived from machine learning algorithms and are not species-specific. These data therefore serve as comparative indicators of potential toxicity and should be interpreted cautiously until validated experimentally. Consistent with the toxicity classification results, ZINC47133693, with an LD_50_ of 10,000 mg/kg, was predicted to be the safest compound, compared to Naproxen and Curcumin, which showed LD_50_ values of 248 mg/kg and 2000 mg/kg, respectively. In contrast, ZINC15942488 was predicted to be the most lethal hit compound, with an LD_50_ of 220 mg/kg. Structural features likely underlie these differences; for instance, reactive or inherently toxic functional groups can increase compound toxicity [42,43]. Comparative studies on structurally related analogs with known LD_50_ values may help identify toxicophores and guide the design of safer derivatives. These findings have important implications for drug development and safety assessment. LD_50_ values and toxicity endpoints indicate that most hit compounds, including Curcumin, are predicted to be non-hepatotoxic, whereas Naproxen, ZINC15942488, ZINC32605424, and ZINC35520239 may pose liver toxicity risks [44]. This likely reflects higher affinity and accumulation in liver cells, including hepatocytes, Kupffer cells, stellate cells, sinusoidal endothelial cells, and cholangiocytes [45,46,47].

Carcinogenicity testing is essential to identify potential tumorigenic risks, especially for compounds structurally related to known carcinogens or with mutagenic properties [48,49]. In this study, ZINC15942488, ZINC47133693, and ZINC47133699 were predicted to be carcinogenic, warranting further investigation. These combined findings underscore the importance of early computational toxicity assessments, including LD_50_, hepatotoxicity, and carcinogenicity, in guiding the selection and optimization of lead compounds for safe and effective drug development Immunotoxicity assessment is essential to identify unintended adverse effects on the immune system that could compromise drug safety, reduce the efficacy of vaccines or other therapies, and increase the risk of infections, autoimmune disorders, or hypersensitivity reactions [50]. Regulatory guidelines, such as the ICH S8, emphasize immunotoxicity testing to ensure that pharmaceuticals do not cause immunosuppression or immune enhancement, which may pose significant health risks, particularly in vulnerable populations. This evaluation informs risk–benefit decisions and supports regulatory approval by providing essential safety data on immune system impacts [51,52]. In this study, Curcumin, ZINC09499196, and ZINC35520239 were predicted to be immunotoxic, suggesting potential interference with normal immune function and increased susceptibility to infections or immune-related adverse effects. Therefore, caution should be exercised when considering these compounds for therapeutic use, and further detailed immunological studies are warranted to fully characterize their effects on immune health.

Cytotoxicity assessment serves as a crucial early screening tool to detect any direct toxic effects of compounds on living cells, helping to identify substances that may cause cellular damage or death before advancing to more complex biological evaluations or clinical trials [53]. This testing is essential for establishing the safety profile of drug candidates, revealing potential harmful effects on cell viability, and ensuring compliance with regulatory requirements such as ISO 10993-5 and FDA guidelines [54]. Cytotoxicity data also inform dose optimization, safety profiling, and risk–benefit assessments during drug development and regulatory submissions. In this study, all compounds were inactive for cytotoxicity except ZINC32605424, whose cytotoxicity suggests a potential unfavorable safety profile in terms of direct cellular toxicity. Therefore, the results for hits such as ZINC47133707 and ZINC26976295, which exhibit low-toxicity classification, high LD_50_ values, and inactivity across the evaluated toxicity endpoints, are encouraging and suggest that these compounds are unlikely to cause cellular damage.

### 2.5. Assessing the Pharmacokinetic Profile of Naproxen, Curcumin, and Selected Hit Compounds

Understanding pharmacokinetics helps predict how the drug behaves in the body, enabling the identification of candidates with favorable bioavailability and minimal toxicity risks, thereby reducing late-stage failures due to poor pharmacokinetic profiles [55]. Moreover, early pharmacokinetics and physicochemical screening support dose optimization, personalized therapy, and the anticipation of drug–drug interactions, all of which are essential for successful clinical outcomes and regulatory approval. These properties critically influence the absorption, distribution, metabolism, and excretion of a drug, which in turn determines its efficacy, safety, and optimal dosing regimen in patients [56]. The pharmacokinetic parameters, including lipophilicity based on iLogP, water solubility based on ESOL, gastrointestinal absorption, P-glycogen protein substrate, and cytochrome P450 isoenzymes, were assessed in this study (see Table 3). Lipophilicity indicates the hydrophobic character of drugs and plays a crucial role in their ADME profile by enhancing membrane permeability and facilitating absorption through passive diffusion [57], with a favorable range between −0.4 and +5.6 [58].

In this study, all compounds were predicted to be within this range. Although Naproxen showed the lowest lipophilicity value of 1.94, hits such as ZINC32605424 and ZINC35520239 had the highest lipophilicity of 4.45 and ZINC26976295 demonstrated the lowest lipophilicity of 2.79, among other hits. These results suggest that ZINC32605424 and ZINC35520239 may facilitate transport of the molecules across the cell membrane lipid bilayer but typically compromise aqueous solubility and can reduce oral absorption. Therefore, increases in LogP should be interpreted cautiously, as excessive lipophilicity generally results in poor dissolution and limited bioavailability. On the other hand, the lower lipophilicity of ZINC26976295 suggests improved water solubility, which may lead to decreased absorption despite favorable solubility-related properties. In terms of water solubility, Curcumin and ZINC09499196 were predicted to be more soluble with water solubility values of −3.94 and −3.80 mg/mL, respectively. Meanwhile, ZINC32605424, ZINC47133707, and ZINC35520239 were predicted to be partially water soluble with solubility values of −7.25, −7.23, and −7.25 mg/mL. Importantly, compounds that are aqueous soluble have a like chance of dissolving in biological fluids which facilitate their absorption in the body.

Gastrointestinal absorption determines the extent to which an orally administered drug enters systemic circulation, directly impacting its efficacy. Half of the compounds, including Curcumin, Naproxen, ZINC08644750, ZINC09499196, ZINC15942488, and ZINC26976295, showed high gastrointestinal absorption, while ZINC32605424, ZINC47133693, ZINC47133699, ZINC47133702, ZINC47133707 and ZINC35520239 showed low gastrointestinal absorption, indicating that they are insoluble. P-glycoprotein, an efflux transporter expressed in the intestinal lining and other tissues, can limit drug absorption by pumping substrates back into the gut lumen, thereby reducing bioavailability and influencing drug interactions [59]. The inhibition of its activity indicates that drugs are bioavailable, and its induction is associated with drug resistance. In this study, several compounds, including ZINC08644750, ZINC09499196, ZINC15942488, ZINC32605424, ZINC47133699, ZINC47133707, ZINC26976295, and ZINC35520239, were identified as P-glycoprotein substrates, suggesting that their absorption may be compromised due to efflux activity. Notably, compounds such as ZINC32605424 and ZINC35520239, which also exhibited low gastrointestinal absorption, may have limited systemic exposure partly because of P-glycoprotein-mediated efflux. Conversely, our findings show that Naproxen, Curcumin, ZINC47133693, and ZINC47133702 are not P-glycoprotein substrates and demonstrated higher gastrointestinal absorption, indicating a greater potential for effective oral bioavailability. The results also show that most P-glycoprotein substrates tend to have higher lipophilicity and lower water solubility, factors that may contribute to their recognition by P-glycoprotein. Understanding the P-glycoprotein substrate status of these compounds is crucial, as it impacts their pharmacokinetic profiles, potential drug–drug interactions, and overall therapeutic efficacy. Therefore, strategies to overcome P-glycoprotein-mediated efflux, such as formulation modifications or co-administration with P-glycoprotein inhibitors, may be necessary for optimizing the clinical performance of P-glycoprotein substrate drugs.

The cytochrome P450 enzymes are crucial for biotransformation and drug metabolism [60]. CYP3A4, the most abundant isoenzyme in the liver and intestine, metabolizes approximately 30–50% of clinically used drugs and exhibits significant interindividual variability influenced by genetic and environmental factors [61,62]; CYP1A2 metabolizes various drugs and environmental toxins and can be induced or inhibited by other compounds; CYP2D6 is responsible for metabolizing many psychotropic and cardiovascular drugs and shows genetic polymorphisms that affect drug response; and CYP2C9 metabolizes drugs such as warfarin and NSAIDs, with activity variations impacting drug safety and efficacy [63,64,65]. Evaluating these parameters early in drug development is important in predicting oral bioavailability, metabolic pathways, and potential interactions, ultimately guiding the selection of safer and more effective drug candidates. In this study, Naproxen showed no inhibitory activity against any of these CYP isoenzymes, consistent with its well-established metabolic profile and generally low potential for CYP-mediated drug interactions [60]. Notably, all compounds except Naproxen and ZINC26976295 inhibit CYP2C9, indicating a potential risk for drug–drug interactions that could affect drug clearance and safety. Curcumin, ZINC08644750, ZINC09499196, ZINC15942488, ZINC32605424, ZINC47133702, ZINC26976295, and ZINC35520239 were predicted to inhibit CYP3A4. Inhibition of CYP3A4 can lead to elevated levels of co-administered drugs metabolized by this enzyme in the blood plasma, increasing the risk of adverse effects. Furthermore, CYP1A2 inhibition was observed only with ZINC15942488 and ZINC47133702, suggesting a more selective interaction profile for this enzyme. Moreover, CYP2D6 inhibition was limited to ZINC09499196 and ZINC15942488, which is notable given the role of CYP2D6 in metabolizing many psychotropic and cardiovascular drugs and its genetic polymorphism-driven variability in patients. Therefore, these compounds have different functional groups that are very distinct from Curcumin, this suggest that different CYP enzymes may be important to metabolize these drugs

These inhibitory profiles have significant clinical implications. CYP inhibition can cause accumulation of substrate drugs, potentially leading to toxicity or enhanced pharmacological effects, as documented in numerous drug–drug interaction cases. For example, potent CYP3A4 inhibitors like ketoconazole or ritonavir are known to dramatically increase plasma concentrations of CYP3A4 substrates. Similarly, CYP2C9 inhibition can affect anticoagulants such as warfarin, necessitating careful dose adjustments. The selective inhibition patterns observed suggest that some compounds, especially ZINC15942488, which inhibit all four CYP enzymes tested, may carry a higher risk of complex drug interactions and require thorough in vivo evaluation. Conversely, compounds like Naproxen, which do not inhibit these enzymes, may have a safer metabolic profile with lower interaction potential. It is also important to note that the CYP inhibition data presented here are in silico predictions based on molecular descriptors and do not confirm direct enzyme binding or inhibition. These results suggest potential metabolic interactions that warrant further validation through molecular docking, MD simulation, or in vitro enzyme inhibition assays.

### 2.6. Assessment of Molecular Dynamics Simulation of Curcumin, Naproxen, and Selected Hit Compounds

The interaction of a bioactive compound with its target protein often induces conformational changes in the secondary and tertiary structures of the protein, thereby modulating its fundamental functions [66]. Unlike static experimental approaches, molecular dynamics (MD) simulations capture the dynamic behavior of both the ligand and the protein, revealing how the conformation of the protein changes upon ligand binding and how the ligand adjusts within the binding site over time. This dynamic modeling is essential for identifying transient or hidden binding pockets, understanding the flexibility of the protein–ligand complex, and predicting binding stability and affinity with greater accuracy than static models. By simulating atomic movements under physiological conditions, MD simulation provide detailed insights into the thermodynamics and kinetics of drug binding, which are critical for optimizing drug candidates [67,68]. These structural changes form the basis of the therapeutic effects of compounds. To further assess the top 10 hit compounds with high docking score and good drug-likeness, we first analyzed the stability of the COX-2 protein using root mean square deviation (RMSD), followed by an assessment of its flexibility through root mean square fluctuation (RMSF) during interactions with the top 10 hits including Naproxen and Curcumin.

#### 2.6.1. Evaluating COX-2 Protein Structural Stability Using Root Mean Square Deviation Analysis

**Figure 4:**
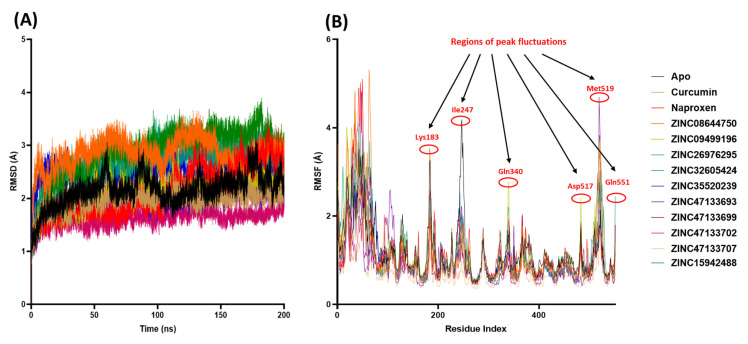
The analysis of the MD simulation of COX-2 in complex with Naproxen, Curcumin, and selected top 10 hits. (**A**) demonstrates the RMSD values of the MD simulated systems, and (**B**) demonstrates the RMSF values of the MD simulated systems. The native COX-2 structure is denoted as Apo.

A 200 ns MD simulation was conducted to investigate the structural dynamics of all systems. Protein convergence and the stability of the MD trajectories were evaluated using RMSD, as illustrated in Figure 4A. Our results show that all systems reached convergence within the first 50 ns, followed by stable atomic motions throughout the entire 200 ns simulation period. Notably, the RMSD values for all systems remained within a narrow range of approximately 1.61 to 2.88 Å, indicating consistent structural stability. This range aligns well with previously reported RMSD values for assessing the molecular stability of COX-2 protein complexes [21,69]. The mean RMSD values (with standard deviations) for the COX-2 systems were as follows: Apo (2.1 ± 0.29 Å), Naproxen (2.12 ± 0.19 Å), Curcumin (1.95 ± 0.47 Å), ZINC08644750 (2.85 ± 0.29 Å), ZINC09499196 (2.12 ± 0.29 Å), ZINC15942488 (2.88 ± 0.49 Å), ZINC26976295 (2.73 ± 0.41 Å), ZINC32605424 (2.61 ± 0.35 Å), ZINC35520239 (2.53 ± 0.19 Å), ZINC47133693 (2.00 ± 0.20 Å), ZINC47133699 (2.02 ± 0.23 Å), ZINC47133702 (1.61 ± 0.15 Å), and ZINC47133707 (2.11 ± 2.11 Å). In general, a desirable RMSD range of approximately 1 to 3 Å is commonly accepted as an indicator of stable protein–ligand complexes during MD simulation [70,71]. These results confirm that all complexes maintained stable conformations during the 200 ns simulation period, supporting their potential as stable COX-2 binding compounds.

#### 2.6.2. Evaluating COX-2 Protein Structural Flexibility Using RMSF Analysis

We employed RMSF analysis to assess the changes in residue-level motion which serves as a measure of the flexibility within specific regions of the COX-2 structure, as illustrated in Figure 4B. The mean RMSF values (with standard deviations) for the COX-2 systems were as follows: Apo (1.22 ± 0.63 Å), Naproxen (1.15 ± 0.72 Å), Curcumin (1.04 ± 0.0.54 Å), ZINC08644750 (1.12 ± 0.77 Å), ZINC09499196 (1.17 ± 0.73 Å), ZINC15942488 (1.19 ± 0.63 Å), ZINC26976295 (1.15 ± 0.60 Å), ZINC32605424 (1.09 ± 0.65 Å), ZINC35520239 (1.08 ± 0.55 Å), ZINC47133693 (1.03 ± 0.60 Å), ZINC47133699 (0.98 ± 0.65 Å), ZINC47133702 (0.96 ± 0.40 Å), and ZINC47133707 (0.68 ± 0.28 Å). An acceptable RMSF value indicating structural stability generally depends on the protein size and context, but fluctuations below approximately 2 Å are widely considered indicative of stable and relatively rigid regions in proteins during MD simulation. Higher RMSF values suggest more flexible or mobile regions, while values under 2 Å typically reflect limited atomic movement and structural stability. This threshold has been reported in several studies analyzing protein flexibility and stability, including those assessing the conformational behavior of enzymes and drug targets like COX-2 [72]. A higher RMSF value typically signifies increased flexibility in the structure, whereas a lower average RMSF value generally reflects a more rigid or less flexible conformation.

As shown in Figure 4B, all the simulated systems showed great amino acid fluctuations within the first 70 amino acid regions of the COX-2. This may indicate structural adaptation of the protein to the simulation environment and interactions with the compounds. Additionally, all simulated systems exhibited prominent fluctuations at COX-2 residues Lys183, Ile247, Gln340, Asp517, Met519, and Gln551—regions that showed the highest mobility during the simulations. Notably, the amino acid residues of the unbound COX-2 protein displayed similar dynamic behavior to those observed in the systems bound with Naproxen, Curcumin, and the top 10 hit compounds throughout the simulation period.

### 2.7. Calculation of the Binding Free Energy of COX-2 and Ligand Complex Using the Molecular Mechanics Generalized Born and Surface Area Approach

The MM/GBSA method is a popular technique for estimating binding energies, known for its greater accuracy compared to most molecular docking scoring functions. Unlike simple docking scores, MM/GBSA incorporates molecular mechanics energies combined with solvation effects, typically derived from MD simulation, which allows it to better capture the physical interactions stabilizing the protein–ligand complex [73]. This leads to improved identification of active and inactive compounds, enhancing the accuracy of virtual screening campaigns. In this study, the MM/GBSA calculations were performed using 200 evenly spaced frames extracted from the final 200 ns trajectories. Although replicate simulations were not conducted, we acknowledge the importance of independent replicates to reduce sampling error and have noted this as a limitation. Binding free energy was calculated, as shown in Table 4.

The results indicate that the ∆G_bind_ range from −31.12 ± 0.25 kcal/mol for Naproxen to −55.70 ± 0.33 kcal/mol for ZINC32605424, indicating a spectrum of interaction strengths with the COX-2 protein. While the ∆E_vdw_ consistently contribute significantly to binding, with values spanning from −35.67 ± 0.16 to −63.23 ± 0.25 kcal/mol. ZINC32605424, which exhibits the strongest overall binding affinity, also shows the most favorable Van der Waals energy (−63.23 ± 0.25 kcal/mol), strengthening the importance of hydrophobic contacts in stabilizing the complex. Furthermore, ∆E_ele_ vary more broadly, from weak interactions such as −2.68 ± 0.25 kcal/mol for ZINC47133693 to substantial contributions like −44.72 ± 0.55 kcal/mol for ZINC32605424, highlighting the ligand-dependent nature of these forces.

The ∆G_gas_, combining Van der Waals and electrostatic terms, is most favorable for ZINC32605424 (−107.96 ± 0.67 kcal/mol) and ZINC08644750 (−90.09 ± 0.88 kcal/mol), although these strong interactions are partially offset by positive ∆G_solv_, reflecting the energetic cost of desolvation upon binding. Despite this penalty, the net binding free energies remain strongly negative for these ligands, indicating robust binding. These findings align with previous studies indicating that Van der Waals and electrostatic energies are primary driving forces in ligand binding, while solvation effects modulate overall affinity [74,75]. In relation to compounds in comparison, Curcumin shows moderate binding affinity (−37.44 ± 0.34 kcal/mol) with balanced Van der Waals and electrostatic interactions, while Naproxen has the weakest binding energy (−31.12 ± 0.25 kcal/mol). Notably, the binding free energy of Curcumin with COX-2 is consistent with values reported in our previous study, reinforcing the reliability of these results [21]. Several ZINC compounds surpass the reference ligands in binding strength, suggesting their potential as more effective binding elements of COX-2 protein. These ΔG_bind_ values represent enthalpic contributions only; full free energies including entropy would require separate normal mode analysis.

However, it is essential to recognize the inherent statistical uncertainties associated with MM/GBSA calculations. Due to factors including limited sampling, force field approximations, and use of implicit solvation models, typical errors can be on the order of 2 to 3 kcal/mol or more. Consequently, differences in binding free energy within this error margin should be interpreted cautiously and are not necessarily indicative of meaningful differences in ligand affinity. To address this limitation, performing multiple replicate MM/GBSA calculations from independent MD simulations is strongly recommended.

These findings emphasize the critical role of Van der Waals forces, supported by electrostatics, in driving ligand binding, with solvation effects modulating overall affinity. While MM/GBSA provides valuable relative binding estimates, it is important to consider its limitations, such as the use of implicit solvation models and the neglect of entropy, which may affect absolute accuracy [74,76]. Overall, the analysis identifies promising ligands with strong binding profiles, offering more understanding for further optimization and experimental validation.

### 2.8. Analysis of Molecular Interactions in Molecular Dynamics Simulation Systems Using LigPlot Analysis

The LigPlot analysis after 200 ns of MD simulation provided detailed virtual screening into the molecular interactions between COX-2 and Naproxen, Curcumin, and the top 10 screened hits. Intermolecular forces such as hydrogen and hydrophobic forces, are crucial in maintaining the stable association between ligands and their target proteins, ensuring effective binding and biological activity. Furthermore, hydrogen bonds and hydrophobic interactions are critical intermolecular forces that play essential roles in stabilizing protein–ligand complexes, making their analysis important in drug candidate screening [77]. Hydrogen bonds provide specificity and directionality to binding by forming strong, localized interactions between polar groups of the ligand and key amino acid residues in the target protein. These bonds help anchor the ligand in the correct orientation within the binding site, enhancing binding affinity and selectivity. Hydrophobic interactions, on the other hand, arise from the tendency of nonpolar ligand regions to associate with hydrophobic pockets on the protein surface, reducing unfavorable water contacts and contributing significantly to the overall binding free energy [77,78].

Together, these interactions define the strength and stability of the ligand–protein complex, influencing both efficacy and specificity of potential drug candidates. As demonstrated by LigPlot analysis after MD simulation in Table 5, mapping these interactions provides a detailed understanding of how ligands engage with COX-2, enabling more accurate prediction of binding stability and guiding rational optimization of compounds. The results in Table 5 indicate that hydrophobic interactions were conserved across all complexes, involving key active site residues such as Leu320, Val317, Ala495, Tyr323, and Phe486. Notably, Tyr323 has been reported as a conserved residue in our previous study, underscoring its important role within the COX-2 binding site. Additionally, residues including Leu320, Val317, and Ala495 were previously identified as primary participants in the interaction between COX-2 and Curcumin [21]. These residues might be strong interacting sites of COX-2 with compounds barring key functional groups found in the structure of Curcumin. These findings suggest that these amino acids serve as critical interaction hotspots for COX-2 binding, not only with Curcumin but potentially with other compounds sharing key functional groups. The conservation of these hydrophobic contacts aligns with established knowledge that the COX-2 active site is predominantly hydrophobic, facilitating ligand stabilization through Van der Waals interactions and contributing to binding specificity and affinity [79]. Thus, these residues likely represent strong interacting sites essential for the effective binding of diverse ligands within the COX-2 active site.

For example, Naproxen formed hydrophobic contacts with residues including Leu320, Phe486, and Met490, complemented by two hydrogen bonds with Tyr353 (2.77 Å) and Tyr323 (2.56 Å), indicating a stable binding mode consistent with its known inhibitory activity. Curcumin exhibited a broader interaction network, engaging hydrophobically with residues like Leu50, Val85, and Tyr84 and forming hydrogen bonds with Tyr316 (3.36 Å) and Lys47 (2.82 Å), which may explain its moderate binding affinity observed in MM/GBSA calculations. Among the top hits, ZINC32605424 demonstrated extensive hydrophobic contacts with residues such as Trp68, Ile81, and Phe486, alongside multiple hydrogen bonds with Val57 (3.02 Å), Arg89 (2.91 Å and 2.94 Å), Ser321 (2.90 Å), and Ser98 (3.00 Å), suggesting a strong and specific interaction pattern that correlates with its favorable binding free energy. Similarly, ZINC26976295 and ZINC08644750 showed multiple hydrophobic interactions and key hydrogen bonds with residues like Tyr353 (3.12 Å), Val491 (3.28 Å), and Arg89 (2.84 Å and 3.11 Å), and Leu320 (2.90 Å), which are critical for COX-2 ligand recognition and inhibition. In contrast, some compounds such as ZINC35520239 and ZINC47133702 lacked hydrogen bonds but maintained numerous hydrophobic contacts, indicating that Van der Waals interactions alone can contribute substantially to binding stability [80].

The presence of conserved hydrogen bonds with residues like Tyr323, Arg89, and Ser321 across several top hits highlights their importance in ligand binding and potential selectivity towards COX-2. These residues have been previously implicated in COX-2 inhibitor binding and catalytic function, underscoring the relevance of the observed interactions. The combination of hydrophobic contacts and hydrogen bonding networks observed in these ligands supports their strong binding affinities and suggests their potential as effective COX-2 inhibitors. Overall, the LigPlot analysis complements the MM/GBSA binding energy results by revealing the specific molecular interactions underpinning ligand binding. The detailed interaction profiles provide valuable structural insights that can guide further optimization of these compounds to enhance potency and selectivity. This integrative approach confirms that both hydrophobic interactions and hydrogen bonds are essential for stable and selective inhibition of COX-2, consistent with previous structural and computational studies on COX-2 inhibitors. A limitation of this study is that COX-1 and COX-2 selectivity was not evaluated. Future work will include cross-docking, binding energy comparison, and MD-based selectivity profiling to determine whether the identified hits preferentially target COX-2, which is essential to reduce gastrointestinal toxicity commonly associated with non-selective NSAIDs.

## 3. Materials and Methods

### 3.1. Retrieval and Preparation of COX-2 Structure

The X-ray crystal structure of COX-2 (PDB ID: 5F1A) with a resolution of 2.38 Å was obtained from the Research Collaboratory for Structural Bioinformatics (RCSB) Protein Data Bank (PDB) in .pdb format [81]. The protein was prepared for molecular docking using University of California, San Francisco (UCSF) Chimera version 1.16 [82] by removing water molecules and non-standard residues as previously described [21]. To validate the inhibitory effects of the identified compounds on COX-2, Naproxen, which is a potent COX-2 inhibitor that is an FDA-approved NSAID, was used as a reference compound in this study [10,83]. Additionally, Curcumin, a potential COX-2 inhibitor, served as a comparator throughout the investigation.

### 3.2. Generating Pharmacophore Models Through Per-Residue Energy Decomposition Approach and Virtual Screening

The Molecular docking and MD simulation of COX-2, Naproxen, and Curcumin complexes were performed in our previous study [21] to interrogate interactions between Curcumin, Naproxen, and COX-2. For molecular docking, Curcumin and Naproxen were docked into COX-2 binding site using AutoDock Vina via the UCSF Chimera interface (version 1.16, build 42360), following standard preparation steps: water molecules, ions, and non-standard residues were removed prior to docking. Docking results guided the binding affinity for COX-2 complexes for further interaction analysis in MD simulation for 200 ns using AMBER, under standard Number of particles, Pressure, and Temperature (NPT) conditions as previously demonstrated [21]. During preparation, the COX-2 structures were optimized, binding sites predicted, and ligand force fields assigned with General AMBER Force Field (GAFF) in AMBER. Per-residue energy decomposition (PRED) was conducted using the MM/GBSA approach on the simulated systems, allowing identification of residue-level interaction energies. This information then informed the creation of a PRED-based pharmacophore model derived from ligand–receptor interactions at stable states in the MD trajectories [84,85]. The model was queried against the ZINCPharmer interface to screen ZINC-22 purchasable compounds with molecular weight ≤ 500 g/mol and rotatable bonds set at ≤10. The “rule of five” proposed by Lipinski served as an additional cut–off [34].

### 3.3. Molecular Docking Assessment of Selected Hit Compounds

A molecular docking study was conducted on the identified hits to assess binding scores and elucidate the optimal ligand–receptor binding orientation with COX-2. Prior to docking, the hit compounds underwent energy minimization using Avogadro 1.2.0 software [86], which employs the Universal Force Field (UFF) to optimize molecular geometries through the steepest descent algorithm. The active site for the COX-2 target was determined using grid positions of Curcumin and Naproxen docking positions that were previously identified using the Computed Atlas of Surface Topography of proteins (CASTp) free webserver [21,87], with the binding site coordinates set at Center (X = 58.20, Y = 44.19, Z = 36.88) and Dimensions (X = 28.61, Y = 18.28, Z = 18.93). Subsequently, molecular docking was performed using AutoDock Vina plugin within PyRx software prescription 0.8 [88] for all hit compounds. The docking results were visualized in UCSF Chimera using the integrated ViewDock module [82]. The docking scores for the best 10 complexes with the 2-Dimensional structures of the compound were denoted, see Figure 2, and saved for subsequent analysis.

### 3.4. Predicting Pharmacokinetic, Physicochemical, and Drug-likeness Properties of Selected Hit Compounds

The selected hit compounds were further analyzed for their physicochemical properties, including absorption, distribution, metabolism, and excretion (ADME). Utilizing the online platform SwissADME (http://www.swissadme.ch/) [89], which predicts and analyzes pharmacokinetic and physicochemical properties of selected compounds, was essential for evaluating the prospects of the identified hits for human use. Furthermore, in silico ADME studies are important to mitigate the risk of late-stage attrition in drug development and optimize screening and testing by focusing on promising compounds [90]. ADME properties were predicted based on Lipinski’s rule of five, a standard for estimating biological activity, good oral bioavailability, and the ability of a drug molecule to penetrate various aqueous and lipophilic barriers [91,92].

### 3.5. Prediction of Toxicological Properties of Hit Compounds

Toxicity prediction for the hit compounds was conducted using the Protox-II server (https://tox-new.charite.de/protox_II/), accessed on: 30 January 2024 [93]. This server enables the quantification of toxicity endpoints such as mutagenicity, carcinogenicity, and other characteristics, both quantitatively and qualitatively, to assess the toxicity of chemical compounds [94].

### 3.6. Molecular Dynamics Simulation to Confirm Interaction Stability of Selected Hit Compounds

The MD simulation provided by the SANDER engine from the AMBER package ((https://ambermd.org), accessed on: 30 January 2024), with the systems described using the AMBER force field FF14SB, was utilized [95]. Atomic partial charges for the ligand were determined using ANTECHAMBER along with the Restrained Electrostatic Potential (RESP) and General Amber Force Field (GAFF) procedures. Protonation states were generated at physiological pH (7.4) using the Leap module in AMBER 14, which automatically added hydrogen atoms and neutralized the system by adding 1 Na^+^ ion to the COX-2 protein. Amino acids were re-numbered according to the dimeric form of the protein, resulting in residues numbered from 1 to 551. The simulation systems were constructed by placing the systems within an orthorhombic box filled with TIP3P water molecules. The positioning ensured that all atoms were within 10 Å of any box edge.

Initially, a minimization process was conducted with a restraint potential of 500 kcal/mol applied to both solutes. This minimization comprised 1000 steps using the steepest descent method, followed by an additional 1000 steps utilizing conjugate gradients. Subsequently, a full minimization of 1000 steps were carried out using the conjugate gradient algorithm without any restraints. The systems underwent gradual heating from 0 K to 310.15 K (37 °C; physiological temperature) over a 0.05 ns MD simulation. Throughout this process, the number of atoms and volume remained constant. The solutes within the systems were subjected to a potential harmonic restraint of 10 kcal/mol and a collision frequency of 0.001 ns. Following the heating phase, an equilibration step of approximately 0.5 ns was conducted for each system, maintaining the temperature at 310.15 K. The systems were simulated under an isobaric–isothermal ensemble, with certain parameters held constant, including the number of atoms and pressure. The pressure of the system was regulated at 1 bar using the Berendsen barostat.

The MD simulation was executed for 200 ns utilizing AMBER 18 GPU version. During each simulation, the SHAKE algorithm was applied to constrain bonds involving hydrogen atoms. A time step of 2 fs was employed, and the simulations utilized an SPFP precision model. Operating under isobaric–isothermal ensemble conditions, the simulations incorporated randomized seeding, maintaining a constant pressure of 1 bar through the Berendsen barostat. The pressure-coupling constant was set at 0.002 ns, and a physiological temperature was maintained using a Langevin thermostat with a collision frequency of 0.001 ns [21].

### 3.7. Analysis of Post-Molecular Dynamics Simulation of Selected Hit Compounds

Following the preservation of the coordinates of all systems, trajectory analysis was carried out at 100 ns intervals using process trajectory (PTRAJ). This analysis encompassed the calculation of RMSD and RMSF, facilitated by the CPPTRAJ module. Molecular interaction analysis was conducted using Molegro Molecular Viewer version 2.2 [96] and LigPlot version 2.2.8 [97] software to delineate ligand binding interactions and intermolecular interactions within the protein’s binding site at the conclusion of the simulation period (200 ns).

### 3.8. Calculating Binding Free Energy Contribution of Selected COX-2 Inhibiting Hit Compounds

To assess and contrast the binding affinity of the systems, the MM/GBSA method [98] was employed to determine the binding free energy. This involved averaging 200 snapshots extracted from the 200 ns trajectory. The resulting binding free energy (ΔG) obtained through this approach [99] for each molecular species (complex, ligand, and receptor) can be delineated as follows:

(1) ΔG_bind_ = G_complex_ − G_receptor_ − G_ligand_
(2) ΔG_bind_ ≈ ΔE_vdw_ + ΔE_ele_ + ΔG_solv_
(3) E_gas_ = E_int_ + E_vdw_ + E_ele_
(4) G_sol_ = GGB + GSA
(5) GSA = ΔSASA

In this equation, the “E_gas_” represents the energy in the gas phase, comprising internal energy (E_int_), Coulomb energy (E_ele_), and Van der Waals energy (E_vdw_). Egas was directly calculated using the FF14SB force field terms. Solvation free energy (G_sol_) was estimated by considering contributions from both polar states (GGB) and non-polar states “G”. The non-polar solvation energy (GSA) was determined based on the solvent accessible surface area (SASA) using a water probe radius of 1.4 Å. Conversely, the polar solvation contribution (GGB) was estimated by solving the Generalized Born (GB) equation. The terms “S” and “T” represent the total entropy of the solute and the temperature, respectively. The MMGBSA calculations omitted the entropic term −TΔS due to high computational cost and convergence challenges in normal mode analysis, providing enthalpic approximations suitable for relative ligand ranking.

### 3.9. Computational Cost and Hardware Details

All simulations were performed using the AMBER 18 GPU version on a NVIDIA Tesla V100 GPU (5120 CUDA cores, 32 GB memory), supported by dual Intel Xeon Gold 6248 processors (40 cores, 2.50 GHz) and 256 GB RAM. Each 200 ns simulation required approximately 72 GPU hours per system, depending on the system size and solvent configuration.

### 3.10. Data Analysis

Visualization of the data was conducted using Discovery Studio Visualizer version 21.1.0.20298 [100] and LigPlot+ version 2.2 [101] software. Figures were generated using GraphPad prism version 8.0.1, Boston, MA, USA, www.graphpad.com declared.

## 4. Conclusions

This study presents a robust in silico pipeline integrating pharmacophore-based screening, molecular docking, ADME profiling, MD simulation, MM/GBSA energy calculations, and LigPlot analysis to identify novel COX-2 inhibitors structurally related to Curcumin. From an initial virtual screen of 237 compounds, 10 promising hits were identified with high docking scores to COX-2, outperforming reference compounds Naproxen and Curcumin. These hits demonstrated excellent geometrical fit within the COX-2 active site and favorable physicochemical and pharmacokinetic properties, with most conforming to drug-likeness criteria. Among these, ZINC32605424 emerged as the top-performing compound, exhibiting the most favorable MM/GBSA binding energy, strong interactions with conserved COX-2 residues (Tyr323, Leu320, Val491), and consistent structural stability over a 200 ns simulation. While a few compounds exhibited limitations such as low solubility (ZINC08644750, ZINC15942488, and ZINC26976295) or synthetic complexity (ZINC47133699 and ZINC47133707), these challenges offer opportunities for future structural optimization. Toxicity assessments highlighted that ZINC47133693 and ZINC09499196 had lower predicted toxicity, with high LD_50_ values and minimal cytotoxic risk. LigPlot analyses underscored the importance of hydrogen bonding and hydrophobic interactions in maintaining ligand stability, while ADME profiling identified potential concerns such as gastrointestinal absorption and CYP enzyme inhibition for a few hits. Compounds such as ZINC32605424, ZINC08644750, ZINC47133693, and ZINC09499196 demonstrate strong potential as COX-2 inhibitors, with improved predicted stability, safety, and pharmacokinetic profiles compared to Curcumin. All ADME and toxicity findings are computational predictions and should be experimentally validated before any biological or clinical inference is made. Therefore, experiments tailored to validate these compounds as potential COX-2 inhibitors are recommended. Our findings provide potential new chemical scaffolds for the development of safer and more effective anti-inflammatory agents.

## Figures and Tables

**Table 1:** The physicochemical and lead-likeness properties of Naproxen, Curcumin and the 10 selected hit Compounds.

Compound	Molecular Weight (g/mol)	Number of Hydrogen Bond Acceptors	Number of Hydrogen Bond Donors	Octanol/Water Partition Coefficient (LogP)	Drug Likeness (Lipinski’s Rule)	Synthetic Score
Naproxen	230.26	3	1	3.04	Yes	1.85
Curcumin	368.38	6	2	3.37	Yes	2.97
ZINC08644750	429.51	3	2	4.76	Yes	3.68
ZINC09499196	464.51	5	2	2.44	Yes	4.05
ZINC15942488	389.45	3	3	4.96	Yes	4.00
ZINC32605424	447.55	4	1	6.90	No	4.07
ZINC47133693	488.65	5	3	4.30	Yes	4.69
ZINC47133699	496.64	5	3	4.54	Yes	4.86
ZINC47133702	496.58	4	3	4.37	Yes	4.29
ZINC47133707	517.06	5	3	4.89	No	4.73
ZINC26976295	410.42	5	3	2.44	Yes	4.07
ZINC35520239	447.55	4	1	6.00	Yes	4.08

**Table 2:** The summary of the toxicity class and endpoints of the selected hit Compounds compared to Curcumin and Naproxen.

Compound	Class	LD_50_ (mg/kg)	Hepatotoxicity	Carcinogenicity	Immunotoxicity	Cytotoxicity
Naproxen	3	248	Active; *P* (0.51)	Inactive; *P* (0.53)	Inactive; *P* (0.85)	Inactive; *P* (0.80)
Curcumin	4	2000	Inactive; *P* (0.61)	Inactive; *P* (0.84)	Active; *P* (0.92)	Inactive; *P* (0.88)
ZINC08644750	4	570	Inactive; *P* (0.68)	Inactive; *P* (0.65)	Inactive; *P* (0.96)	Inactive; *P* (0.66)
ZINC09499196	5	2209	Inactive; *P* (0.82)	Inactive; *P* (0.63)	Active; *P* (0.79)	Inactive; *P* (0.58)
ZINC15942488	3	220	Active; *P* (0.55)	Active; *P* (0.58)	Inactive; *P* (0.98)	Inactive; *P* (0.70)
ZINC32605424	4	2000	Active; *P* (0.60)	Inactive; *P* (0.60)	Inactive; *P* (0.99)	Active; *P* (0.58)
ZINC47133693	6	10,000	Inactive; *P* (0.50)	Active; *P* (0.51)	Inactive; *P* (0.99)	Inactive; *P* (0.61)
ZINC47133699	4	1600	Inactive; *P* (0.50)	Active; *P* (0.50)	Inactive; *P* (0.99)	Inactive; *P* (0.61)
ZINC47133702	4	350	Inactive; *P* (0.50)	Inactive; *P* (0.54)	Inactive; *P* (0.84)	Inactive; *P* (0.61)
ZINC47133707	4	1600	Inactive; *P* (0.51)	Inactive; *P* (0.55)	Inactive; *P* (0.97)	Inactive; *P* (0.63)
ZINC26976295	4	1300	Inactive; *P* (0.51)	Inactive; *P* (0.51)	Inactive; *P* (0.79)	Inactive; *P* (0.53)
ZINC35520239	4	1190	Active; *P* (0.69)	Inactive; *P* (0.62)	Active; *P* (0.96)	Inactive; *P* (0.93)

**Table 3:** Assessment of the administration, distribution, metabolism, and excretion profile of Curcumin, Naproxen, and selected hit Compounds.

Compounds	Lipophilicity (iLogP)	Water Solubility (ESOL, mg/mL)	Gastrointestinal (GI) Absorption	P-Glycoprotein Substrate	CYP3A4	CYP1A2	CYP2D6	CYP2C9
Naproxen	1.94	−3.61	High	No	No	No	No	No
Curcumin	3.27	−3.94	High	No	Yes	No	No	Yes
ZINC08644750	3.33	−5.15	High	Yes	Yes	No	No	Yes
ZINC09499196	3.14	−3.80	High	Yes	Yes	No	Yes	Yes
ZINC15942488	3.46	−5.29	High	Yes	Yes	Yes	Yes	Yes
ZINC32605424	4.45	−7.25	Low	Yes	Yes	No	No	Yes
ZINC47133693	3.48	−6.47	Low	No	No	No	No	Yes
ZINC47133699	3.74	−6.94	Low	Yes	No	No	No	Yes
ZINC47133702	3.69	−6.92	Low	No	Yes	Yes	No	Yes
ZINC47133707	3.67	−7.23	Low	Yes	No	No	No	Yes
ZINC26976295	2.79	−3.61	High	Yes	Yes	No	No	No
ZINC35520239	4.45	−7.25	Low	Yes	Yes	No	No	Yes

**Table 4:** The analysis of the MM/GBSA-based binding free energy contributions of active site residues in the protein–ligand complex.

	Energy Components (kcal/mol)				
	∆E_vdw_	∆E_ele_	∆G_gas_	∆Gs_olv_	∆G_bind_
Naproxen	−35.67 ± 0.16	−9.48 ± 0.30	−45.15 ± 0.32	14.03 ± 0.13	−31.12 ± 0.25
Curcumin	−46.67 ± 0.25	−21.48 ± 0.53	−68.17 ± 0.60	30.73 ± 0.40	−37.44 ± 0.34
ZINC08644750	−57.62 ± 0.27	−32.47 ± 0.79	−90.09 ± 0.88	37.72 ± 0.41	−52.37 ± 0.52
ZINC09499196	−53.17 ± 0.25	−15.66 ± 0.54	−68.83 ± 0.59	24.70 ± 0.30	−44.13 ± 0.38
ZINC26976295	−55.52 ± 0.24	−32.48 ± 0.58	−88.00 ± 0.55	37.22 ± 0.38	−50.80 ± 0.28
ZINC32605424	−63.23 ± 0.25	−44.72 ± 0.55	−107.96 ± 0.67	52.25 ± 0.42	−55.70 ± 0.33
ZINC35520239	−54.41 ± 0.28	−3.58 ± 0.29	−58.00 ± 0.41	14.76 ± 0.24	−43.24 ± 0.27
ZINC47133693	−57.08 ± 0.20	−2.68 ± 0.25	−59.76 ± 0.32	15.12 ± 0.19	−44.65 ± 0.23
ZINC47133699	−60.72 ± 0.28	−5.76 ± 0.40	−66.48 ± 0.36	14.40 ± 0.29	−52.08 ± 0.25
ZINC47133702	−57.39 ± 0.20	−5.23 ± 0.38	−62.62 ± 0.42	14.29 ± 0.21	−48.32 ± 0.27
ZINC47133707	−56.17 ± 0.23	−7.38 ± 0.44	−63.55 ± 0.43	14.76 ± 0.28	−48.79 ± 0.26
ZINC15942488	−55.98 ± 0.23	−23.42 ± 0.39	−78.40 ± 0.42	32.02 ± 0.27	−46.39 ± 0.27

∆E_vdw_—change in Van der Waals energy; ∆E_ele_—change in electrostatic energy; ∆G_gas_—change in Gibbs free energy in the gas phase; ∆Gs_olv_—change in Gibbs free energy of solvation; ∆G_bind_—change in Gibbs free energy of binding.

**Table 5:** LigPlot analysis of molecular interactions between COX-2 protein and Naproxen, Curcumin, and the Top 10 hits after 200 ns of simulation.

Compound Name	Hydrophobic Interactions	Hydrogen Bond (Length-Å)
Naproxen	Leu320, Phe486, Met490, Val491, Ala495, Ser498, and Gly494	Tyr353 (2.77) and Tyr323 (2.56)
Curcumin	Leu50, Val85, Lys51, Arg89, Ser88, Tyr84, Leu499, Ser498, Trp355, Gly494, and Leu320	Tyr316 (3.36) and Lys47 (2.82)
ZINC08644750	Ala495, Val317, Arg89, Pro52, Val85, Phe325, Val57, Pro54, Thr53, Ser321, Tyr323, Phe486, Val491, Met490, and Ser498	Leu320 (2.90)
ZINC09499196	Ile50, Val57, Ser321, Ala495, Tyr353, Leu320, Phe74, Phe86, Val317, Tyr323, Leu61, and Val85	Arg89 (2.81 and 2.99) and Ser489 (2.75)
ZINC26976295	Leu320, Val317, Gly494, Ser498, Ser321, Thr56, Pro52, Tyr84, Val85, Phe325, Ala495, and Tyr323	Tyr353 (3.12), Val491 (3.28), and Arg89 (2.84 and 3.11)
ZINC32605424	Trp68, Ile81, Phe326, Val317, Leu499, Leu320, Met490, Gly494, Ala495, Val491, Phe486, Val85, and Leu61	Val57 (3.02), Arg89 (2.91 and 2.94), Ser321 (2.90) and Ser98 (3.00)
ZINC35520239	Trp68, Phe325, Ser321, Val85, Tyr84, Tyr323, Leu50, Ile81, Pro52, Hie324, and Ile60	No hydrogen bonds
ZINC47133693	Ala495, Tyr353, Ser321, Val317, Leu320, Tyr84, Ile81, Val57, Val85, and Val491	Tyr323 (3.01) and Arg481 (2.84)
ZINC47133699	Val491, Ser321, Ala484, Leu61, Val85, Val57, Tyr323, Gly494, Ser498, Tyr353, Ala495, and Leu320	Arg481 (2.84), Hie58 (3.11), and Arg89 (3.16)
ZINC47133702	Phe74, Val317, Tyr316, Val312, Ala495, Leu499, Ser321, Tyr323, Ile81, Trp68, Val57, Leu327, Val491, and Met490	No hydrogen bonds
ZINC47133707	Arg89, Phe326, Ile81, Trp68, Leu61, Val85, Hie58, Ser321, Val491, Ala495, SER498, and Val317	Tyr323 (3.05)
ZINC15942488	Leu61, Ile60, Ile81, Val85, Leu327, Phe325, Met82, Hie58, Val317, Ser498, Gly494, Tyr353, Leu320, Phe486, Val491, and Val57	Ser321 (2.88)

## Data Availability

The original contributions presented in this study are included in the article. Further inquiries can be directed to the corresponding author.
